# Characterization of the complete mitochondrial genome and phylogenetic status of a recently described species of Mountain Dragon, *Diploderma vela* (Reptilia: Squamata: Agamidae), from the upper Lantsang valley in west China

**DOI:** 10.1080/23802359.2021.1962756

**Published:** 2021-09-27

**Authors:** Yayong Wu, Ke Li, Feng Wang, Qin Liu, Bo Cai

**Affiliations:** a Faculty of Agriculture, Forest and Food Engineering, Yibin University, Yibin, China; b Forestry and Grassland Bureau of Aba, Aba, China; c College of Life Science, Shenyang Normal University, Shenyang, China; d Museum of Herpetology, Chengdu Institute of Biology, Chengdu, China

**Keywords:** Lizard, mitochondrial genome, mountain dragon, phylogeny, topotype

## Abstract

The mountain dragon, *Diploderma vela*, is an endemic and protected valley lizard that inhabits the upper Lantsang Valley in West China. In this study, we sequenced the complete mitochondrial genome of a male individuals of *D. vela* using next-generation sequencing methodologies. The complete mitogenome is 16,432 bp in length and contains one noncoding control regions, 13 protein-coding, 22 transfer RNA and two ribosomal RNA genes. The mitogenome content and structure of D. vela was consistent with the previously published representatives of the family. A Bayesian phylogenetic analysis using the complete mitochondrial genomes of Agamidae fully resolved *D. vela* in the Draconinae, a result consistent with previous investigations. This study provides bioinformatic data for better understanding the evolution and the phylogenetic history of the mountain dragon.

The mountain dragon, *Diploderma vela*, is an endemic valley lizard that inhabits elevations about 2300 m along the upper Lantsang valley in West China (Wang et al. 2015). Unfortunately, the native plant communities and physical structures of valley habitats are being damaged by human activities, such as agricultural planting, road construction and local townships expansion (Wang et al. 2019a). These activities threaten the conservation of this endemic species. This species was recently listed as one of the second-class protected animals of China in early 2021. Meanwhile, our limited knowledge of *D. vela* has extremely restricted the establishment of conservation strategies and measures (Wang et al. 2019b; Uetz et al. 2020). In this study, we describe the complete mitochondrial genome of *D. vela* obtained from next-generation sequencing methods and analyze its phylogenetic relationships with other members within the Agamidae.

A male specimen (CIB5421290081) of *D. vela* was collected from the type locality of Quzika of Markam county (29°05'N, 98°36'E), Tibet province, China in July 2018. The specimen was identified based on Wang et al. (2015). The specimen and the liver tissue (fixed with 95% ethanol, −20°C) were deposited in the herpetological collection, Chengdu Institute of Biology, Chinese Academy of Science (http://herpmuseum.cib.ac.cn, Li Jia-Tang, lijt@cib.ac.cn). Total genomic DNA was extracted from liver tissue using Trelief Animal Genomic DNA Kit (Tsingke, Beijing, China) following the manufacturer’s instruction with minor modification. The complete mitochondrial DNA sequence was analyzed on an Illumina HiSeq 2000 platform. Genes were assembled and annotated with the SPAdes v3.11.0 (Bankevich et al. 2012) and MITOS web server (Bernt et al. 2013), respectively. The mitogenome was submitted to GenBank under the accession number MW788326. All sampling activities were conducted in accordance with the Guidelines of Animal Ethics published by the Chengdu Institute of Biology.

The complete mitochondrial genomes of *D. vela* was 16,432 bp in length, comprising one non-coding control region (CR), 13 protein-coding genes (PCGs), two ribosomal RNA genes, and 22 transfer RNA genes (tRNA), while lacking origin of light-strand replication (OL). The mitogenome base-pair is AT biased (58.8%) with 34.8% for A, 28.1% for C, 13.1% for G and 23.9% for T. Most genes were located on the heavy strand (H-strand) with the exception of *ND6* and eight tRNA genes (*tRNA-Gln, Ala, Asn, Cys, Tyr, Ser^UCN^, Glu,* and *Pro*). The mean length of tRNA genes was 68 bp, the shortest and the longest were *tRNA-Cys* gene (54 bp) and *tRNA-Leu* (75 bp), respectively. The mean length of PCGs was 865 bp, the shortest and the longest were ATP8 gene (162 bp) and ND5 (1779 bp), respectively. Most PCGs initiated with ATG except for *ATP8*, and *ND5*, both started with GTG. Seven PCGs terminated with complete stop codons, TAA (*ND4L*, *ND5*), AGG(*ND1*), AGA (*COX1*), CAC (*ATP8*), and TAG (*ND2*, and *ND6*), while the other six genes ended with the incomplete stop codon, TA/T (*COX2*, *ATP6*, *COX3*, *ND3*, *ND4*, and *Cytb*). The mitogenome content and structure of *D. vela* was consistent with the previously published representatives of the family (Liu et al. 2019; Huang et al. 2019; Li et al. 2021).

Phylogenetic analysis based on nucleotide sequences of 13 PCGs of *D. vela* with the other 17 species of Agamidae, both Uromastycinae (*Uromastyx benti*) and Leiolepidinae (*Leiolepis belliana*) were designated as outgroups based on published higher-level phylogenetic studies of squamate reptiles (Pyron et al. 2013). Bayesian phylogenetic tree using the GTR + I + G substitution model indicated that *D. vela* was closely related to its congeners, fully resolved in the subfamily Draconinae (PP 1.00) (Figure 1). The overall phylogenetic relationships among Agamidae were consistent with previous studies (Wang et al. 2019a). This study provides a valuable mitogenome resource for better understanding the molecular evolution and phylogenetic relationships of *D. vela*, and serves as a reference for the establishment of conservation strategies and measures.

**Figure 1.**
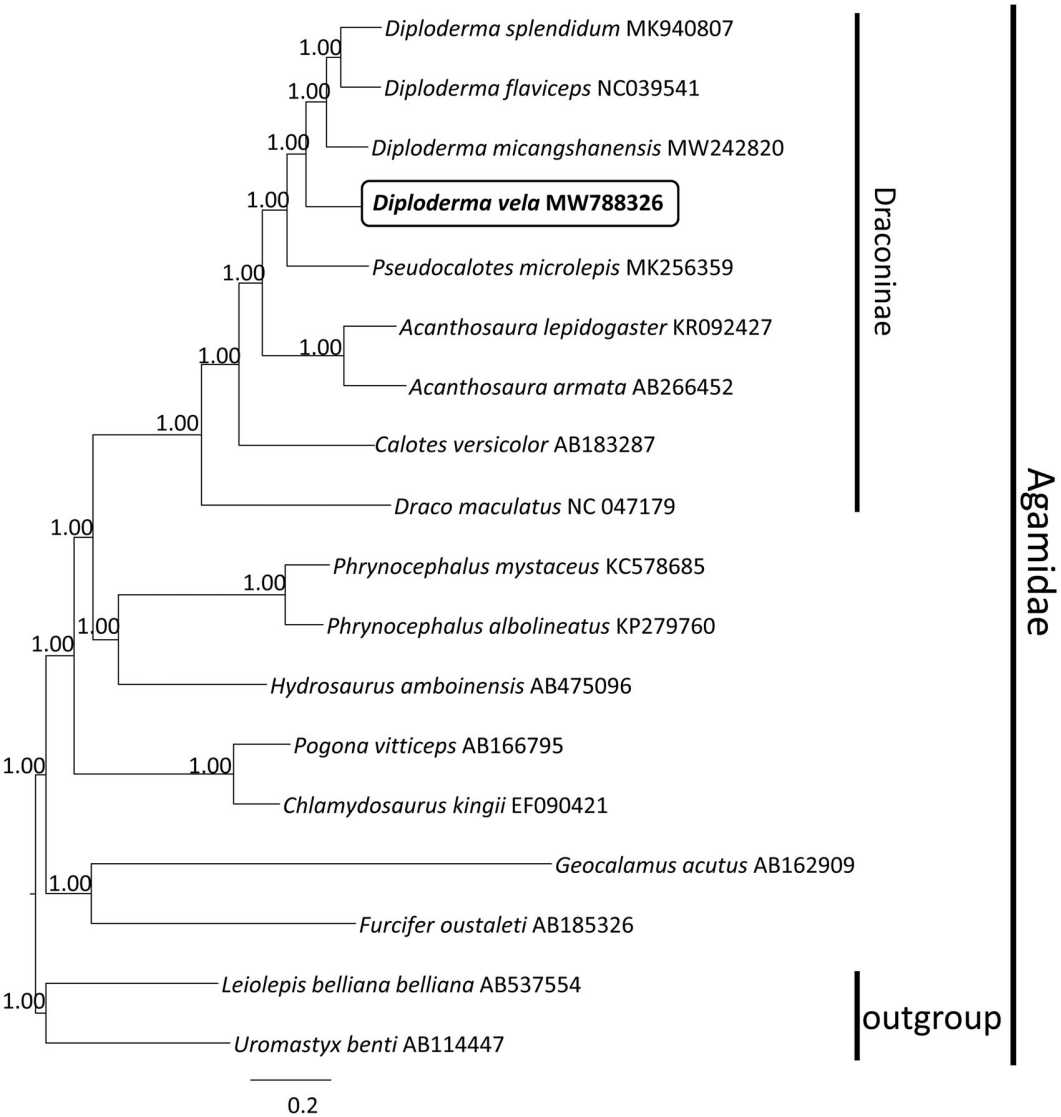
Majority rule consensus tree of PCGs of 18 species of Agamidae inferred using MrBayes v.3.2.2 (Ronquist et al. 2012) with a GTR + I + G substitution model selected by MrModelTest 2.3 (Nylander 2004) under the Akaike information criterion. DNA sequences were aligned in MEGA 7 (Kumar et al. 2016). Node numbers show Bayesian posterior probabilities. Branch lengths represent means of the posterior distribution. GenBank accession numbers are given with species names.

## Data Availability

The data that support the findings of this study are openly available in NCBI (National Center for Biotechnology Information) with GenBank Accession No. MW788326 (https://www.ncbi.nlm.nih.gov/nuccore/MW788326) and DRYAD (Dryad Digital Repository) with the unique DOI (doi:10.5061/dryad.qjq2bvqgb).
